# PIRCHE-II Is Related to Graft Failure after Kidney Transplantation

**DOI:** 10.3389/fimmu.2018.00321

**Published:** 2018-03-05

**Authors:** Kirsten Geneugelijk, Matthias Niemann, Julia Drylewicz, Arjan D. van Zuilen, Irma Joosten, Wil A. Allebes, Arnold van der Meer, Luuk B. Hilbrands, Marije C. Baas, C. Erik Hack, Franka E. van Reekum, Marianne C. Verhaar, Elena G. Kamburova, Michiel L. Bots, Marc A. J. Seelen, Jan Stephan Sanders, Bouke G. Hepkema, Annechien J. Lambeck, Laura B. Bungener, Caroline Roozendaal, Marcel G. J. Tilanus, Joris Vanderlocht, Christien E. Voorter, Lotte Wieten, Elly M. van Duijnhoven, Mariëlle Gelens, Maarten H. L. Christiaans, Frans J. van Ittersum, Azam Nurmohamed, Junior N. M. Lardy, Wendy Swelsen, Karlijn A. van der Pant, Neelke C. van der Weerd, Ineke J. M. ten Berge, Fréderike J. Bemelman, Andries Hoitsma, Paul J. M. van der Boog, Johan W. de Fijter, Michiel G. H. Betjes, Sebastiaan Heidt, Dave L. Roelen, Frans H. Claas, Henny G. Otten, Eric Spierings

**Affiliations:** ^1^Laboratory of Translational Immunology, University Medical Center Utrecht, Utrecht University, Utrecht, Netherlands; ^2^PIRCHE AG, Berlin, Germany; ^3^Department of Nephrology, University Medical Center Utrecht, Utrecht University, Utrecht, Netherlands; ^4^Laboratory Medicine, Laboratory Medical Immunology, Radboud University Medical Center, Nijmegen, Netherlands; ^5^Department of Nephrology, Radboud University Medical Center, Nijmegen, Netherlands; ^6^Julius Center for Health Sciences and Primary Care, University Medical Center Utrecht, Utrecht University, Utrecht, Netherlands; ^7^Division of Nephrology, Department of Internal Medicine, University Medical Center Groningen, University of Groningen, Groningen, Netherlands; ^8^Department of Laboratory Medicine, University Medical Center Groningen, University of Groningen, Groningen, Netherlands; ^9^Department of Transplantation Immunology, Tissue Typing Laboratory, Maastricht University Medical Center, Maastricht, Netherlands; ^10^Central Diagnostic Laboratory, Division of Immunology, Maastricht University Medical Center, Maastricht, Netherlands; ^11^Department of Internal Medicine, Division of Nephrology, Maastricht University Medical Center, Maastricht, Netherlands; ^12^Department of Nephrology, VU University Medical Center, Amsterdam, Netherlands; ^13^Department of Immunogenetics, Sanquin, Amsterdam, Netherlands; ^14^Renal Transplant Unit, Department of Internal Medicine, Academic Medical Center (AMC), Amsterdam, Netherlands; ^15^Dutch Organ Transplant Registry (NOTR), Dutch Transplant Foundation (NTS), Leiden, Netherlands; ^16^Department of Nephrology, Leiden University Medical Center, Leiden, Netherlands; ^17^Department of Internal Medicine, Erasmus MC, Rotterdam, Netherlands; ^18^Department of Nephrology, Erasmus MC, Rotterdam, Netherlands; ^19^Department of Immunohematology and Blood Transfusion, Leiden University Medical Center, Leiden, Netherlands

**Keywords:** PIRCHE-II, kidney transplantation, graft rejection, HLA antigens, HLA matching

## Abstract

Individual HLA mismatches may differentially impact graft survival after kidney transplantation. Therefore, there is a need for a reliable tool to define permissible HLA mismatches in kidney transplantation. We previously demonstrated that donor-derived Predicted Indirectly ReCognizable HLA Epitopes presented by recipient HLA class II (PIRCHE-II) play a role in *de novo* donor-specific HLA antibodies formation after kidney transplantation. In the present Dutch multi-center study, we evaluated the possible association between PIRCHE-II and kidney graft failure in 2,918 donor–recipient couples that were transplanted between 1995 and 2005. For these donors–recipients couples, PIRCHE-II numbers were related to graft survival in univariate and multivariable analyses. Adjusted for confounders, the natural logarithm of PIRCHE-II was associated with a higher risk for graft failure [hazard ratio (HR): 1.13, 95% CI: 1.04–1.23, *p* = 0.003]. When analyzing a subgroup of patients who had their first transplantation, the HR of graft failure for ln(PIRCHE-II) was higher compared with the overall cohort (HR: 1.22, 95% CI: 1.10–1.34, *p* < 0.001). PIRCHE-II demonstrated both early and late effects on graft failure in this subgroup. These data suggest that the PIRCHE-II may impact graft survival after kidney transplantation. Inclusion of PIRCHE-II in donor-selection criteria may eventually lead to an improved kidney graft survival.

## Introduction

Kidney transplantation is the preferable treatment option for most patients suffering from end-stage kidney disease. Kidney transplantation outcome is most optimal when patient and donor are HLA matched (1, 2). HLA mismatches may lead to the activation of alloreactive T-cells and the development of donor-specific HLA antibodies (DSA), thereby significantly impairing kidney graft survival (3). In HLA-mismatched kidney transplantation graft survival decreases gradually with increasing numbers of mismatches on HLA-A, HLA-B, and HLA-DRB1 (2, 4). Although most research has been devoted to studying the impact of HLA-A, -B, and -DRB1 mismatching on kidney graft survival, some studies suggest that HLA-C and HLA-DQB1 may impact graft survival as well (5, 6). Since the role of HLA-A, HLA-B, and HLA-DRB1 has been extensively described, the European organ exchange organization Eurotransplant has implemented an algorithm in which currently the number of HLA-A, HLA-B, and HLA-DRB1 mismatches is taken into consideration in the allocation strategy.

HLA-matching implies that better matched kidney grafts (0–3 mismatches at HLA-A, -B, and -DRB1) are preferred over poorly matched kidney grafts (4–6 mismatches at HLA-A, -B, and -DRB1) (4). Since this number of HLA-A, -B, and -DRB1 mismatches is generally calculated using low-resolution HLA typing (4), the actual number of HLA mismatches may even be higher at high-resolution level. These allelic disparities between donor and recipient may contribute to clinically relevant alloimmune responses (4). Moreover, in the current allocation strategy, mismatches at HLA-A, -B, and -DRB1 are regarded equally important. Cumulating evidence, however, suggests that each HLA mismatch may contribute with a different weight to graft survival; some HLA mismatches seem to be more permissible/acceptable than others (7, 8). Thus, a better definition of the acceptable mismatches will improve graft survival.

Immunological graft rejection may originate from allo-specific T-cells or antibodies. T-helper cells play a role in both processes; on one hand, CD4+ T-helper cells can provide help to CD8+ cytotoxic T-cells, thereby facilitating graft-directed CD8+ T-cell responses. However, the mechanism of CD4+ T-cells in providing help to CD8+ T-cells remains elusive (9, 10). On the other hand, CD4+ T-helper cells play an essential role in HLA-specific antibody formation *via* B-cell activation and IgM-to-IgG isotype switching. During this process, mismatched HLA is internalized by B-cells, intracellularly processed, and HLA-derived epitopes can subsequently be loaded onto HLA class II molecules on the surface of B-cells. T-helper cells recognizing these HLA-derived epitopes drive B-cell differentiation and IgM-to-IgG isotype switching (11, 12). Thus, the production of HLA-specific IgG antibodies requires the activation of B-cells by T-helper cells. In this process, the T-helper cell and the B-cell recognize different epitopes derived from the same mismatched HLA molecule, a phenomenon called linked recognition (13).

T-helper cell activation is antigen-specific. In HLA-mismatched transplantation, these T-helper cells likely recognize mismatched HLA epitopes in the context of HLA class II. The Predicted Indirectly ReCognizable HLA Epitopes presented by recipient HLA class II (PIRCHE-II) algorithm is able to predict such HLA-mismatch derived T-cell epitopes by quantifying the number of mismatched donor HLA-derived peptides that can be presented on recipients’ HLA class II molecules, designated as PIRCHE-II (14–16). Several studies have shown that the number of PIRCHE-II is related to HLA-antibody formation after pregnancy (15), pancreas and pancreatic islets transplantation (16), and kidney transplantation (17, 18). Moreover, PIRCHE-II may prime CD4+ T-helper cells that can provide help to cytotoxic CD8+ T-cells as described previously. As such, PIRCHE-II may affect graft rejection *via* both pathways. To determine whether the PIRCHE-II algorithm might refine the definition of permissible HLA mismatches in kidney transplantation, we here investigated the role of PIRCHE-II in kidney graft failure. This study is part of the Dutch PROCARE consortium which analyzes the outcome of all kidney transplantations that were performed in the Netherlands between 1995 and 2005 and aims for improved matching algorithms in kidney transplantation.

## Materials and Methods

### Study Population

**Figure 1:**
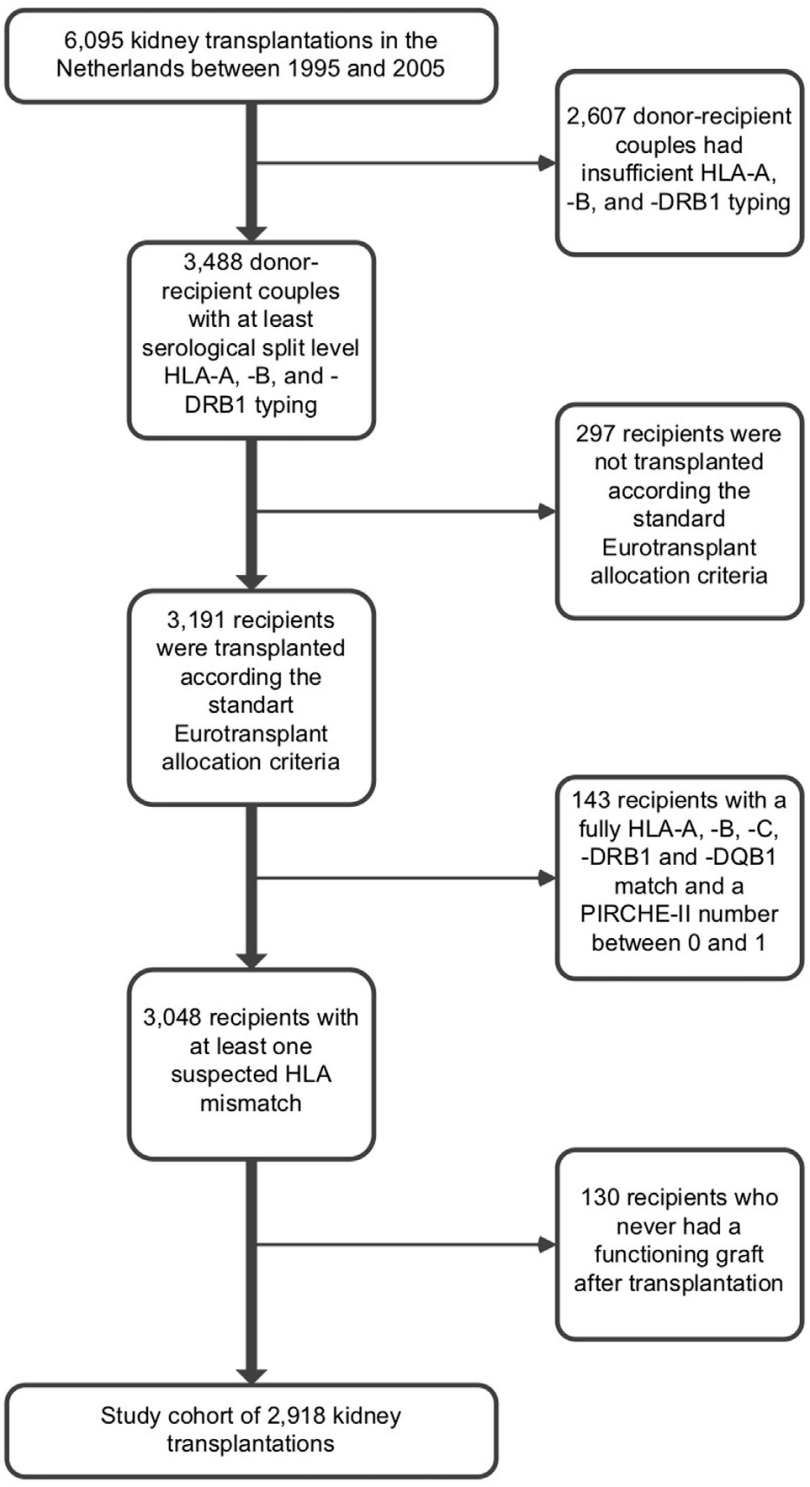
Flow chart of enrollment, inclusion, and exclusions for the study.

This retrospective cohort study included all kidney transplantations that were performed between 1995 and 2005 in the seven different Dutch transplantation centers. The flow chart of enrollment, inclusion, and exclusions for the study is depicted in Figure 1. At time of analysis, HLA typing was available for the majority of 6,095 kidney transplantations that were included in this study. For 3,488 donor–recipient couples, typing resolution was at serological split level or higher for HLA-A, -B, and -DRB1; pairs with lower resolution levels of HLA typing or no typing results for one or more of these loci were excluded from analyses. When HLA-C and HLA-DQ (beta) typing at serological split level was known, the typing results for these loci were also used as input for the PIRCHE-II and HLAMatchmaker algorithms, as described in the next sections. When HLA-C and HLA-DQ (beta) typing was not available at serological split level, typings for these loci were extrapolated using the method described in the next section.

Donor–recipient couples with five or more mismatches at HLA-A, -B, and -DRB1 had an aberrant graft survival compared with other donor–recipient couples, suggesting that the allocations within this group were not performed according the standard Eurotransplant allocation criteria. Since we cannot correct for protocols performed outside the standard Eurotransplant allocation criteria, we excluded these donor–recipient couples to avoid bias (*n* = 297). Donor–recipient couples which were fully matched at serological split level HLA-A, -B, -C, -DRB1, and -DQB1 and had a PIRCHE-II number between 0 and 1 (*n* = 143) were excluded from further analyses, as these couples are most likely to be fully matched at allelic-resolution level. These donor–recipient couples lack an immunological target within donor HLA and thus may have a better graft survival. Inclusion of these donor–recipient couples may therefore enlarge the PIRCHE-II effect observed in this study. Data regarding graft failure, death with functioning graft, and follow-up time was documented. The etiology of graft failures and the presence of DSA before transplantation were not documented. Recipients who never had a functioning graft after transplantation (*n* = 130) were excluded from analyses, resulting in a final cohort size of 2,918 kidney transplantations. Informed consent for evaluation of clinical data was obtained from all subjects involved in the study. The use of clinical data of the subjects involved in this study was approved by the ethics committee for biobanks at the UMC Utrecht (TCBio; reference number: 13-633).

### High-Resolution Extrapolation Method

Since allelic-resolution HLA typing data are unavailable for our cohort, we used the computational method described previously to calculate PIRCHE-II and eplet values using low-resolution HLA typing (19). Briefly, HLA haplotype frequency tables were used to identify the most probable allelic-resolution HLA typings from each donor and recipient low-resolution HLA typing (19, 20). A single low-resolution HLA typing of donor and recipient may result in multiple potential allelic-resolution HLA typings. The PIRCHE-II and eplet values for all these potential allelic-resolution HLA typings were calculated as described in the next sections. Subsequently, the PIRCHE-II and eplet values were weighted by multiplying the normalized frequency of a certain allelic-resolution HLA typing of the recipient in the population by the normalized frequency of a certain allelic-resolution HLA typing of the donor in the population. Finally, all weighted PIRCHE-II and eplet values were summed up, resulting in a decimal PIRCHE-II/eplet number.

### Identification of HLAMatchmaker Eplets

HLAMatchmaker was used to identify three-dimensional polymorphic amino acid patches on the molecular surface of the HLA molecule to which HLA antibodies can be directed, designated as eplets (21–23). The number of HLAMatchmaker eplets was determined for each donor–recipient couple using HLAMatchmaker algorithm version 2.1 (available *via* http://www.epitopes.net/); both antibody-verified and non-antibody-verified eplets were taken into account. In the analyses, eplets that were present in the donor’s HLA and absent in the recipient’s HLA were counted as mismatched eplets. The number of mismatched eplets was determined *via* interlocus HLA comparisons.

### Identification of PIRCHE-II

For all HLA mismatches, we determined the number of donor mismatched HLA-derived peptides that can be presented by recipient HLA-DRB1 (PIRCHE-II) as described previously (15). Briefly, the NetMHCIIpan 3.0 algorithm was used to predict the nonameric binding cores of donor mismatched HLA-derived peptides that can bind to recipient HLA-DRB1. Relevant HLA-DRB1 binders were defined as peptides with an IC_50_ < 1,000 nM for HLA-DRB1 (17, 24). Donor-derived HLA class II binders that differed at least one amino acid in their nonameric binding core with the HLA sequence of the recipient were counted as PIRCHE-II. In the identification of PIRCHE-II, specific donor-epitope-HLA complexes that were present multiple times in a certain donor–recipient couple were counted as a single PIRCHE-II. The PIRCHE algorithm is available *via* https://www.pirche.org.

Since the entire amino acid sequence of relevant HLA molecules is required for the PIRCHE-II algorithm, incomplete amino acid sequences of relevant HLA molecules that were present in the IMGT/HLA database were extrapolated using a nearest-neighbor-based approach as described previously to obtain the complete amino acid sequences of the incomplete HLA allele (25). In our analyses, leader peptide sequences were omitted from the HLA amino acid sequences used for PIRCHE-II calculation.

### Statistical Analysis

Statistical analyses were performed using SPSS Statistics software version 21 (IBM SPSS Software). The time-dependent association of the HLA mismatch-related factors (i.e., the total number of HLA-A, -B, and -DRB1 mismatches, the number of mismatched eplets, and PIRCHE-II) and other covariates (donor age, recipient age, transplantation year, previous transplantations, and recipient immunization status) on graft function was univariately studied with Cox proportional hazard models. Recipient immunization status was based on the maximum panel reactive activity (PRA) percentage measured before transplantation. PRA was determined by a complement dependent cytotoxicity (CDC) test against minimal 50 different donors using the basic NIH technique on unseparated peripheral blood mononuclear cells, which serves as a surrogate marker for the presence of pretransplant cytotoxic DSA. CDC was performed in the presence of Dithiothreitol to prevent IgM-induced false-positive results. A PRA% of 5 was used as cutoff to distinguish between immunized and non-immunized recipients, according our generally used immunization definitions. Multivariable Cox proportional hazard models were constructed to identify variables that impact the 10-year graft survival. The models were constructed using forward stepwise selection considering all univariately analyzed covariates of *p* < 0.10. Except for previous transplantations and recipient immunization status, all covariates were used as continuous variables. For a subgroup of patients who had their first transplantation, additional hazard ratios (HRs) per PIRCHE-II were calculated at different endpoints/time points using forward stepwise selection considering the covariates implemented in the initial multivariable model. Both lost to follow-up and death with functioning graft were considered as censoring events in the analyses. *p*-Values <0.05 were considered statistically significant.

## Results

### Baseline Characteristics

The total analyzable cohort consisted of 2,918 donor–recipient couples. Table 1 summarizes the baseline characteristics of the included donor–recipient pairs. HLA-C and -DQ typing was available for the majority of the donor–recipient couples (HLA-C: 78.2% for donors, 94.9% for recipients and HLA-DQB1: 92.5% for donors, 98.4% for recipients). The median follow-up time of recipients without graft failure during follow-up was 11 years. A total of 675 recipients (23.1% of total) experienced kidney graft failure during follow-up.

**Table 1:** Baseline characteristics (*n* = 2,918).

Median recipient age, years (range)	47 (2–80)
Median donor age, years (range)	46.5 (0–79)
Recipient gender Female, *n* Male, *n*	
	1,196 (41.0%)
	1,722 (59.0%)
Donor gender Female, *n* Male, *n*	
	1,466 (50.2%)
	1,452 (49.8%)
Recipients with prior kidney transplantation(s), *n* 1 prior kidney transplantation, *n* 2 prior kidney transplantations, *n* 3 prior kidney transplantations, *n* 4 prior kidney transplantations, *n*	414
	335
	65
	12
	2
% recipients with HLA-C typing available	94.9%
% recipients with HLA-DQ typing available	98.4%
% donors with HLA-C typing available	78.2%
% donors with HLA-DQ typing available	92.5%
Median broad HLA mismatches (A/B/DR), *n* (IQR) Median broad HLA-A mismatches, *n* (IQR) Median broad HLA-B mismatches, *n* (IQR) Median broad HLA-DR mismatches, *n* (IQR)	2 (1–3)
	1 (0–1)
	1 (0–1)
	1 (0–1)
Delayed graft function, *n*	541 (18.5%)
Graft loss during follow-up, *n*	675 (23.1%)
Median follow-up time, years (IQR)	11 (8.5–14)

### The Number of HLA-A/-B/-DRB1 Mismatches Is Associated with Graft Survival

To confirm the role of HLA mismatches on transplant outcome in our cohort, we first investigated the relationship between the total number of HLA-A, -B, and -DRB1 mismatches and kidney graft survival. HLA-C and HLA-DQ mismatches were not included in these calculations, as HLA-C and HLA-DQ typing was not mandatory according the Eurotransplant guidelines in the years 1995–2005. In univariate analysis, the total number of HLA-A, -B, and -DRB1 mismatches was significantly associated with an increased risk for graft failure (HR: 1.11; 95% CI: 1.04–1.18, *p* = 0.002, Table 2).

**Table 2:** Univariate and multivariable hazard ratios (HRs) of graft failure for ln(PIRCHE-II), eplets, number of HLA-A, -B, and -DR mismatches, age, year of transplantation, retransplantation, and recipient immunization status in the overall cohort.

	Univariate			Multivariable model with forward stepwise selection		
	HR	95% CI	*p*-Value	HR	95% CI	*p*-Value
ln(PIRCHE-II)	1.15	1.06–1.25	0.001	1.13	1.04–1.23	0.003
Eplets	1.01	1.00–1.01	0.01			
Number of A/B/DR mismatches	1.11	1.04–1.18	0.002			
Recipient age	0.99	0.99–1.00	0.003	0.99	0.99–1.00	0.002
Donor age	1.02	1.02–1.03	<0.001	1.02	1.02–1.03	<0.001
Transplantation year	0.98	0.96–1.00	0.06	0.97	0.95–0.99	0.015
Previous transplantations	1.62	1.34–1.95	<0.001	1.56	1.29–1.89	<0.001
Recipient immunization status	1.16	0.99–1.35	0.06			

*A multivariable model with forward stepwise selection was generated using the univariate factors. Only ln(PIRCHE-II), recipient age, donor age, year of transplantation, and retransplantation were included in the model. Eplets, number of HLA-A, -B, and -DR mismatches, and recipient immunization status were not included in the multivariable model and, therefore, no HRs were reported for these factors*.

### PIRCHE-II Is an Independent Risk Factor for Graft Failure

Next, we investigated whether HLA mismatch-related factors and a set of other covariates have an influence on kidney graft failure. The number of PIRCHE-II was log-transformed for the analyses, designated as ln(PIRCHE-II), as PIRCHE-II is logarithmically correlated with HLA-antibody formation (17). In univariate analysis, ln(PIRCHE-II) was significantly associated with an increased risk of graft failure (HR: 1.15, 95% CI: 1.06–1.25, *p* = 0.001; Table 2). Also the number of mismatched eplets was associated with an increased graft failure risk and of the other covariates, only recipient and donor age and previous transplantations were associated with graft failure (Table 2).

**Figure 2:**
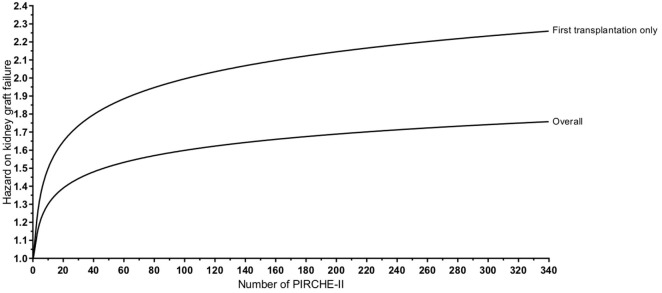
Modeling of the increased hazard on graft failure for each Predicted Indirectly ReCognizable HLA Epitopes presented by recipient HLA class II (PIRCHE-II). Based on the multivariable analyses, the kidney graft failure risk is plotted as a function of the number of PIRCHE-II for both the overall cohort and for the first-transplantations only group. For example for the overall cohort, a patient with a PIRCHE-II value of 2.718 [ln(2.718) = 1] has a hazard of 1.13 on kidney graft failure, whereas a patient with a PIRCHE-II value of 7.388 [=2.718*2.718; ln(7.388) = 2] has a hazard of 1.26 (an increase of 0.13 compared with a PIRCHE-II value of 2.718) on kidney graft failure. Similar calculations were performed for other PIRCHE-II values and for the first-transplantations only group. The differences in hazard on kidney graft failure between different PIRCHE-II values decrease for higher PIRCHE-II values.

To further investigate the possible association between PIRCHE-II and kidney graft failure, a multivariable Cox regression model was constructed considering all univariately analyzed covariates, which allows adjustment for confounders. Recipient and donor age, transplantation year, previous transplantations, and ln(PIRCHE-II) were included in the final model (Table 2). The total number of HLA-A, -B, and -DRB1 mismatches, the number of mismatched eplets, and recipient immunization status did not end up in the multivariate model. ln(PIRCHE-II) was significantly associated with a higher risk of graft failure (HR: 1.13, 95% CI: 1.04–1.23, *p* = 0.003; Table 2). According to this multivariable model, for each increase of one unit ln(PIRCHE-II), which is equivalent to a 2.718-fold difference in PIRCHE-II, the risk of graft failure increases with 13%. Based on this multivariable analysis, we plotted kidney graft failure risk as a function of the number of PIRCHE-II (Figure 2). We observe a non-linear dose-dependent influence of the number of PIRCHE-II on graft failure risk. Thus, PIRCHE-II is an independent risk factor for graft failure after kidney transplantation.

Since the presence of preformed DSA in our study cohort may impact our results, we next analyzed a subgroup of patients who had their first transplantation (*n* = 2,504). Since previous transplantations are a major cause of having pretransplant DSA, patients who have their first transplantation have a reduced chance to have clinically relevant pretransplant HLA alloimmunization. In patients who had their first transplantation, ln(PIRCHE-II) was associated with a higher risk of graft failure (HR: 1.22, 95% CI: 1.10–1.34, *p* < 0.001; Table 3). In these first-transplantation patients, the HR of graft failure for PIRCHE-II was higher compared with the overall cohort (Figure 2), suggesting that PIRCHE-II has a higher impact on graft failure in first-transplantation patients.

**Table 3:** Univariate and multivariable hazard ratios (HRs) of graft failure for ln(PIRCHE-II), eplets, number of HLA-A, -B, and -DR mismatches, age, year of transplantation, and recipient immunization status in the group of first transplantations only.

	Univariate			Multivariable model with forward stepwise selection		
	HR	95% CI	*p*-Value	HR	95% CI	*p*-Value
ln(PIRCHE-II)	1.23	1.11–1.35	<0.001	1.22	1.10–1.34	<0.001
Eplets	1.01	1.00–1.02	0.002			
Number of A/B/DR mismatches	1.15	1.07–1.24	<0.001			
Recipient age	1.00	0.99–1.00	0.053	0.99	0.99–1.00	0.013
Donor age	1.02	1.01–1.03	<0.001	1.02	1.02–1.03	<0.001
Transplantation year	0.97	0.95–1.00	0.048	0.96	0.94–0.99	0.005
Recipient immunization status	0.99	0.83–1.19	0.92			

*A multivariable model with forward stepwise selection was generated using the univariate factors. Only ln(PIRCHE-II), recipient age, donor age, and year of transplantation were included in the model. Eplets, number of HLA-A, -B, and -DR mismatches, and recipient immunization status were not included in the multivariable model and, therefore, no HRs were reported for these factors*.

### PIRCHE-II Demonstrates Early and Late Effects on Graft Failure

**Figure 3:**
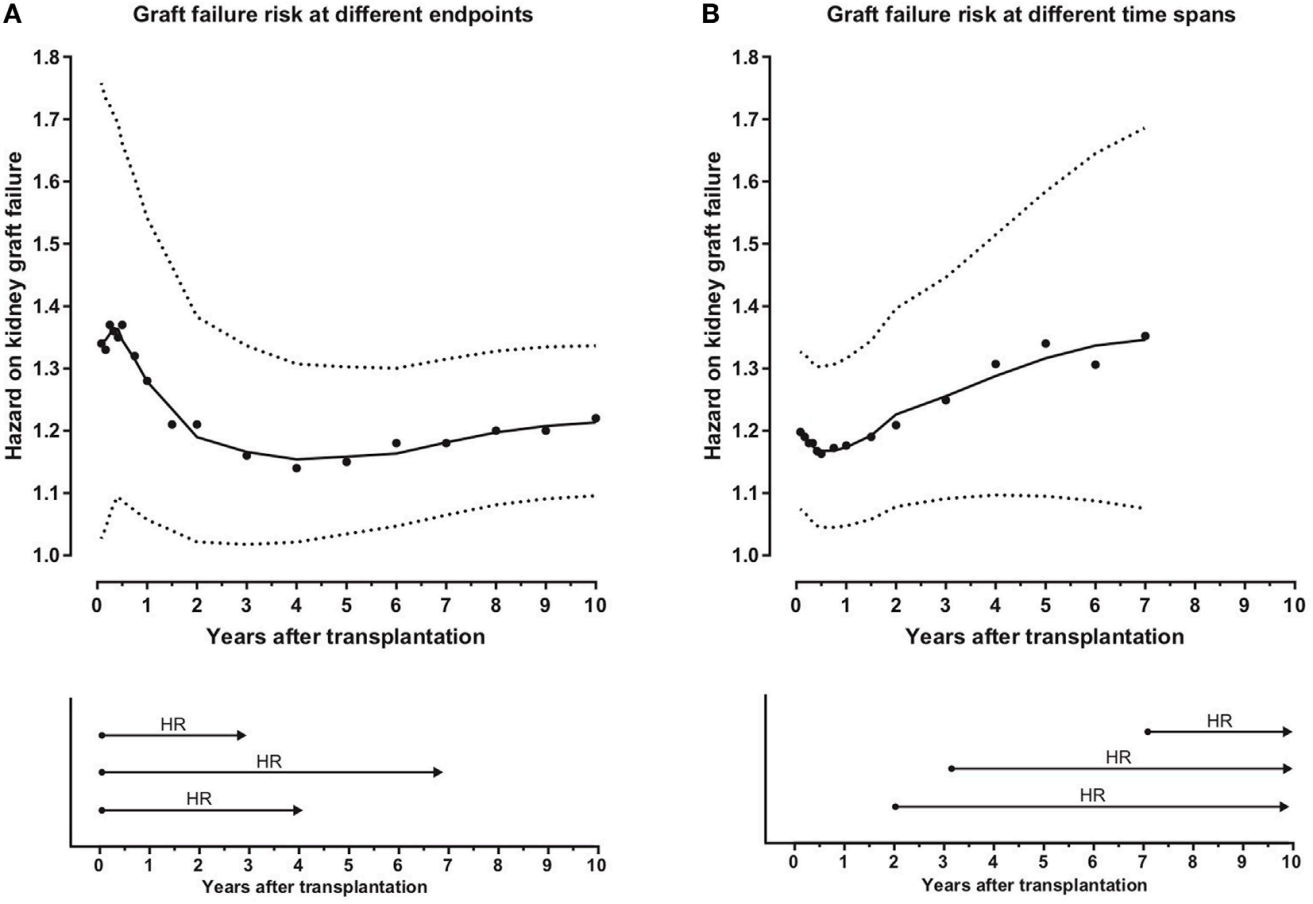
Predicted Indirectly ReCognizable HLA Epitopes presented by recipient HLA class II (PIRCHE-II) affects both early and long-term graft outcome. The hazard on kidney graft failure for ln(PIRCHE-II) at different virtual endpoints after transplantation is displayed in **(A)**. Hazard ratios (HRs) and 95% CI’s were calculated for different virtual endpoints (1–2–3–4–5–6–9–12–18 months and 2–3–4–5–6–7–8–9–10 years after transplantation), as exemplified by the lower panel of **(A)**. HRs were calculated using multivariable Cox proportional hazard models and smooth curve fitting was performed based on these data points. In the upper panel of **(A)**, the solid line represents the HR and the dotted line represents the 95% CI. The kidney graft failure risks for different time spans when excluding subjects who experienced kidney graft failure before different time points are displayed in **(B)**. For these analyses, the graft survival was set to 100% at each analyzed time point (1–2–3–4–5–6–9–12–18 months and 2–3–4–5–6–7 years after transplantation) and the HR were calculated at 10 years after transplantation, as exemplified in the lower panel of **(B)**. The number of patients at risk at each analyzed time span is shown in Table S1 in Supplementary Material. Due to the low number of events and the limited follow-up time around 7 years after transplantation, the later time spans could not be analyzed. HR’s and 95% CI’s were calculated for different time spans using multivariable Cox proportional hazard models and smooth curve fitting was performed based on these data points. In the upper panel of **(B)**, the solid line represents the HR and the dotted line represents the 95% CI. For both **(A,B)**, the individual dots represent the HR’s at different virtual endpoints/time spans. HR’s were calculated using forward stepwise selection considering the covariates implemented in the initial multivariable model [ln(PIRCHE-II), donor age, recipient age, and year of transplantation].

Next we investigated whether PIRCHE-II affects early or long-term graft outcome in multivariable analyses. First we calculated the graft failure risk of ln(PIRCHE-II) at different virtual endpoints after transplantation in the subgroup of patients who had their first transplantation. The HR per ln(PIRCHE-II) peaks around 6 months after transplantation (HR: 1.37, 95% CI: 1.10–1.71), and afterward the HR per ln(PIRCHE-II) remains stable around 1.2 per ln(PIRCHE-II) (Figure 3A). Additionally, we investigated the kidney graft failure risk of ln(PIRCHE-II) at different time points after transplantation among subjects who did not experience graft loss before those different time points. Thus, for each time point, the graft survival was set to 100% and subsequently the HR was calculated. After an initial dip in the first half year, the kidney graft failure risk of ln(PIRCHE-II) slightly increases over time (Figure 3B). Thus, the effect of PIRCHE-II on kidney graft failure is most prominent around 6 months after transplantation (Figure 3A). Our data also suggest that, in patients who do not reject their graft during this early period after transplantation, PIRCHE-II numbers remain associated with a higher kidney graft failure during the following years (Figure 3B). Thus, PIRCHE-II seems to have long-term effects on transplant outcome independent of the early effects.

## Discussion

In the present study, we investigated whether the PIRCHE-II is related to graft survival after kidney transplantation. The total number of HLA-A, -B, and -DRB1 mismatches was associated with kidney graft survival. This observation is in line with previous observations showing that kidney graft survival gradually decreases when transplanted with increasing numbers of HLA mismatches (1, 2). We also showed that increasing PIRCHE-II numbers are associated with a higher risk for kidney graft failure. In the stepwise forward multivariable Cox regression model, PIRCHE-II appeared to be an independent risk factor for kidney graft failure. A stepwise backward multivariable Cox regression model showed a similar result (data not shown). Moreover, PIRCHE-II demonstrated both early and late effects on graft failure. Thus, our data support the hypothesis that the number of PIRCHE-II is indeed related to graft failure after kidney transplantation and confirm the observations of an independent parallel study (17). We therefore conclude that the PIRCHE-II classification may be a tool to identify permissible HLA mismatches in kidney transplantation.

Pretransplant cytotoxic DSA may have affected our observations. In the absence of pretransplant DSA data, we performed a subanalysis in patients who had their first transplant and, consequently, are less likely to have pretransplant DSA. These patients showed an increased HR for kidney graft failure when compared with the overall cohort, suggesting that previous transplants, as a pseudo marker for pretransplant DSA, impact the effect of PIRCHE-II. Similar observations were seen when comparing male and female subgroups separately (data not shown). Whether these latter observations indeed indicate a role for pregnancy-induced DSA or not, remains to be elucidated; alternative gender-related issues may influence these data. Additional research and validations of the pregnancy results in other cohorts are warranted.

Our study has several limitations. First, in the multivariable analysis we included HLA-mismatch-related confounders and a number of additional confounders. These additional confounders were selected based on their available at time of analysis; only confounders that were validated and reliably available for most of the included donor–recipient couples were included in the analyses. However, also other factors, for example, immunosuppressive treatment regimen, etiology of graft loss, cold-ischemia time, type of donor, and transplantation center might have their impact on graft failure. Since some of these data are currently being retrospectively collected for this PROCARE cohort, further studies are required to investigate the role of PIRCHE-II on graft failure in a detailed covariate analysis. Second, our current PIRCHE-II algorithm is restricted to PIRCHE-II derived from HLA-A, -B, -C, -DRB1, and -DQB1 and presented by HLA-DRB1. However, PIRCHE-II might also be derived from other HLA alleles and presentation of PIRCHE-II by other HLA class II alleles may occur. For a major part of our study cohort, HLA typing of these other HLA loci is lacking. For the donor–recipient couples with HLA typing of these additional HLA loci available, we were unable to extrapolate high-resolution HLA typings of these loci due to the absence of these loci in the 2007 HLA haplotype frequency tables (26). Further research in different study cohorts is required to investigate whether addition of PIRCHE-II presented by or derived from these additional HLA loci may further improve the definition of permissible HLA mismatches. Third, further research on IC_50_ cutoff values may eventually lead to a more optimal method to define relevant HLA class II binders.

The development of *de novo* HLA-antibody formation is highly associated with a reduced 10-year graft survival (3, 27). We previously showed that PIRCHE-II is related to HLA-antibody formation (15, 16, 18). The number of PIRCHE-II indeed increased the chance of developing DSA and impaired kidney graft failure (17), suggesting that PIRCHE-II at least partly affects kidney graft function indirectly *via* HLA-antibody formation. In our cohort, we were unable to investigate the effect of PIRCHE-II on HLA-antibody formation, since sera were not collected after transplantation. In addition to driving HLA-antibody formation, PIRCHE-II might also induce graft failure by providing T-cell help to cytotoxic T-cells. Further research is required to investigate whether donor kidney HLA-derived PIRCHE-specific T-cells may indeed also impact kidney graft function without intervention of the B-cell compartment.

The PIRCHE-II associated effect on graft failure is more prominent in the present study than in the study of Lachmann et al. (17), which might be due to differences in donor type, immunosuppressive treatment regimen, or additional factors such as cold-ischemia time between both studies. Moreover, a different HLA-antibody screening procedure after transplantation and subsequent adaptation of the treatment regimen might also explain the graft survival differences. In the Dutch transplantation centers, regular HLA-antibody monitoring were not part of the routine follow-up between 1995 and 2005, but was performed prospectively in the German cohort. In the latter cohort, the immunosuppressive treatment regimen was adapted (intensified or changed) when HLA antibodies were detected, which might have reduced the risk for graft failure. Although future research is required to investigate the effect of immunosuppression adaptation after HLA-antibody detection on graft failure, this difference between both studies might explain the difference in magnitude of PIRCHE-II on graft failure.

High-resolution HLA typings were not yet available for our cohort. Therefore, we used a validated HLA extrapolation method to identify all possible allelic-resolution HLA typings for a given low-resolution HLA typing. We previously showed that PIRCHE-II and eplet numbers can reliably predicted for the majority of the donor–recipient couples using our method (19). The removal of HLA-C or HLA-DQ from the input slightly diminished the reliability of the PIRCHE-II and eplet estimations (19), indicating that the absence of HLA-C/HLA-DQ only limitedly affect the PIRCHE-II values. A subanalysis among donor–recipient couples having complete HLA-A, HLA-B, HLA-C, HLA-DR, and HLA-DQ typing available showed a similar hazard on kidney graft failure for PIRCHE-II as the total cohort (HR: 1.13, 95% CI: 1.02–1.23, *p* = 0.02), indicating that the absence of HLA-C and/or HLA-DQ typing in a minor part of our cohort did not influence the study outcome. Although our approach may have resulted in an over- or underestimation of the exact PIRCHE-II/eplet numbers for certain recipient–donor combinations, the used multiple imputation approach is likely more reliable than selecting a single high-resolution HLA typing according to the most frequent allelic-resolution HLA haplotype in the population; our method does not exclude other potential allelic-resolution HLA haplotypes and the data were analyzed at population level, not at individual donor–recipient level. Importantly, since our multiple imputation approach has been validated in a Caucasian setting, further validation of the approach is warranted before implementing this approach in cohorts with a different ethnic background.

The study outcome might be biased by basing the HLA locus mismatch assignment on HLA-A, -B, and -DRB1 alone, as these typings were available for all donor–recipient couples. Ideally, HLA-C and -DQ also need to be included in the mismatch assignment. However, determining the number of HLA-C and -DQ mismatches *via* the extrapolation method used in our study may result in unreliable HLA mismatch assignment. As HLA-C and -DQ typing is now mandatory according the Eurotransplant guidelines, future studies will be able to show the impact of these loci. Further studies will also be required to investigate the impact of locus-specific PIRCHE-II scores on *de novo* DSA formation, as this may explain differential impact of individual HLA mismatches on graft failure. In the study of Lachmann et al. (17), the role of locus-specific PIRCHE-II scores on DSA formation was investigated. However, due to the absence of post-transplant DSA data, we were unable to validate these observations in our study.

The number of mismatched eplets is highly associated with DSA formation (18, 21, 27–29). Additionally, increasing numbers of HLA-A and HLA-B HLAMatchmaker triplet mismatches are associated with decreasing graft survival rates in HLA-DR identical kidney transplants (23). Based on these data, we implemented HLAMatchmaker eplets as covariate in the analyses in order to avoid overestimation of the PIRCHE-II effect on graft failure. However, in this study we did not aim to compare HLAMatchmaker and the PIRCHE-II algorithm with regard to performance. Detailed research is required to investigate this aspect and also to determine whether a further refinement of the immunogenicity of individual eplets may improve the predictability of kidney graft failure in our cohort. Alternative approaches for estimating the humoral response toward HLA mismatches, such as the number of disparate amino acids or the physicochemical disparities (29), will be included in these detailed future analyses.

In conclusion, our study showed that PIRCHE-II is associated with graft failure after kidney transplantation. We showed that PIRCHE-II might be a more gradual discriminator in defining the permissibility of HLA mismatches. In the future, the PIRCHE-II algorithm might be a manner to identify mismatches that will have a reduced risk for kidney graft failure. Incorporating the PIRCHE-II algorithm in the donor-selection procedure for kidney transplantation may facilitate risk classification of specific mismatches and eventually lead to an improved graft survival. PIRCHE-II may be used in the donor-selection procedure in two ways. First, PIRCHE-II can be used to select the most optimal donor, thereby replacing the currently used HLA matchgrade and match probability. Second, in, for example, recipients receiving grafts from living donors, the PIRCHE-II algorithm can also be used for assessing the risk for graft failure before transplantation. Patients who are at higher risk for kidney graft failure due to a high PIRCHE-II score might benefit from more intense immunosuppression or can be monitored more closely after transplantation.

## Ethics Statement

This study was approved by the ethics committee for biobanks at the UMC Utrecht (TCBio; reference number: 13-633). All patients have consented in participation in the registry and the use of these data in anonymized research. The consortium has received permission to use this data from the management committee of the NOTR (the Dutch Organ Transplant Registry).

## Author Contributions

All authors met the authorship criteria as described by *Frontiers in Immunology*. KG, MN, JD, and ES were involved in design of the work and interpretation of the data. AZ, IJ, WA, AM, LH, MB, CH, FR, MV, EK, MLB, MS, JS, BH, AL, LB, CR, MT, JV, CV, LW, ED, MG, MC, FI, AN, JL, WS, KP, NW, IB, FB, AH, PB, JF, MGHB, SH, DR, FC, and HO were involved in acquisition of the data. All authors were involved in drafting or revising the manuscript and approved the final version. All authors agree to be accountable for all aspects of the work in ensuring that questions related to the accuracy or integrity of any part of the work are appropriately investigated and resolved.

## Conflict of Interest Statement

The authors of this manuscript have conflicts of interest to disclose. The UMC Utrecht has filed a patent application on the prediction of an alloimmune response against mismatched HLA. ES is listed as inventor on this patent. MN is employed by PIRCHE AG, that publishes the PIRCHE web-portal. The other authors declare no conflicts of interest with regard to this publication.

## Abbreviations

DSA, donor-specific HLA antibodies; PIRCHE-II, predicted indirectly recognizable HLA epitopes presented by HLA class II; PRA, panel reactive activity.
