# Supplementary material for: PIRCHE-II Is Related to Graft Failure after Kidney Transplantation
Source: Front Immunol. 2018 Mar 5;9:321. doi: 10.3389/fimmu.2018.00321 (PMC5844930; doi:10.3389/fimmu.2018.00321)
Supplement: Supplementary file 1 [file Table_1.PDF]

Supplementary table 1: Number at risk

Time after transplantation	*Number at risk*
0 months	2,504
1 month	2,415
2 months	2,381
3 months	2,350
4 months	2,335
5 months	2,326
6 months	2,319
9 months	2,288
12 months	2,259
16 months	2,219
2 years	2,166
3 years	2,058
4 years	1,973
5 years	1,865
6 years	1,757
7 years	1,666
